# Influence of the Electron Beam and the Choice of Heating Membrane on the Evolution of Si Nanowires’ Morphology in In Situ TEM

**DOI:** 10.3390/ma15155244

**Published:** 2022-07-29

**Authors:** Ya Shen, Xuechun Zhao, Ruiling Gong, Eric Ngo, Jean-Luc Maurice, Pere Roca i Cabarrocas, Wanghua Chen

**Affiliations:** 1 School of Physical Science and Technology, Ningbo University, Ningbo 315211, China; 2011077022@nbu.edu.cn (Y.S.); 2111077058@nbu.edu.cn (X.Z.); 1911077011@nbu.edu.cn (R.G.); 2 Laboratoire de Physique des Interfaces et des Couches Minces (LPICM), Centre National de la Recherche Scientifique (CNRS), Ecole Polytechnique, Institut Polytechnique de Paris, 91128 Palaiseau, France; eric.ngo@c2n.upsaclay.fr (E.N.); jean-luc.maurice@polytechnique.edu (J.-L.M.); pere.roca@polytechnique.edu (P.R.i.C.)

**Keywords:** electron beam irradiation, silicon nanowire, growth, TEM

## Abstract

We used in situ transmission electron microscopy (TEM) to observe the dynamic changes of Si nanowires under electron beam irradiation. We found evidence of structural evolutions under TEM observation due to a combination of electron beam and thermal effects. Two types of heating holders were used: a carbon membrane, and a silicon nitride membrane. Different evolution of Si nanowires on these membranes was observed. Regarding the heating of Si nanowires on a C membrane at 800 °C and above, a serious degradation dependent on the diameter of the Si nanowire was observed under the electron beam, with the formation of Si carbide. When the membrane was changed to Si nitride, a reversible sectioning and welding of the Si nanowire was observed.

## 1. Introduction

Low-dimensional materials such as nanoparticles, nanotubes, nanowires (NWs), and graphene attract strong interest because of their unique properties as compared to bulk materials. To open up all of these unique properties, a deep understanding of the materials themselves is extremely important. Advanced characterization of the materials is indispensable for their development and optimization. Among these advanced characterizations, different techniques have been developed targeting different properties. For example, atom probe tomography (APT), which is a three-dimensional (3D) characterization technique based on a projection microscope combined with a time-of-flight mass spectrometer, is widely used in fine compositional (100 parts per million) and spatial (Angstrom) characterization of advanced materials [1,2]. Although the 3D information of APT is very useful, the observation of material evolution in real time is also very important. To integrate the fourth dimension of time into APT would be impossible, since APT is a destructive technique that relies on the evaporation of atoms from the sample. Fortunately, many other techniques can be applied as a function of time, so-called in situ techniques, including in situ X-ray diffraction, in situ transmission electron microscopy (TEM) and in situ scanning tunneling microscopy. Among all of these in situ techniques, in situ TEM is most widely used due to its various configurations. Several types of in situ TEM have been developed, such as in situ heating TEM [3,4,5], in situ cryo TEM [6,7], in situ gas environmental TEM [8,9,10,11,12], in situ liquid TEM [13,14], in situ nanomechanical TEM [15], etc. In situ heating TEM, obtained by integrating a heating holder into a TEM analysis chamber, has been widely used in metal and semiconductor science. The holder can be heated by a current via the Joule effect or by a laser. Therefore, it can be used to obtain a better understanding of the fundamental physical phenomena associated with NW or nanotube materials [16]. In situ TEM can be used to observe the dynamic changes of NWs [16,17,18], control their growth [19,20,21,22,23], and induce the formation of nanotubes [24,25] and nanopores [26]. For example, Harmand et al. observed the atomic layer nucleation by growing GaAs NWs in environmental TEM [27]. Yuan et al. used environmental TEM to observe the dissolution and regrowth dynamics of MoO_2_ NWs [28]. Kohno et al. used in situ TEM to observe the transformation of a SiC NW into a carbon nanotube via Joule heating [25]. Wen et al. grew axial heterojunction silicon–germanium NWs by tuning the growth parameters in in situ TEM [21].

Since in situ TEM is dedicated to observing the real-time material evolution, the influence of the electron beam (e-beam) cannot be ignored [1,2]. It has been reported that the e-beam can cause increased dislocation activation, marked stress relaxation [29], anomalous sample necking [30], and amorphization due to inelastic scattering of electrons [31,32]. The e-beam can also induce recrystallization [33,34]. On the other hand, when e-beam irradiation and heating coexist, the degradation of the NWs is more prone to occur, and is more severe [35]. However, all of the above studies only mentioned that the temperature change induced by the electron beam is negligible, and there are few papers investigating the synergistic effects of the electron beam and temperature on the morphology of Si NWs. Moreover, electron irradiation and thermal energy can affect the atomic diffusion, induce nanoparticles’ migration and coalescence, and may contribute to the growth of nanoparticles [36]. When energetic electrons penetrate a solid, they undergo not only inelastic scattering with atomic electrons—which results in the excitation or ejection of electrons—but also elastic scattering with target nuclei that results in knock-on displacements of target atoms [37]. In this work, in situ heating TEM was used to study the influence of electron irradiation on the morphology of Si NWs. Moreover, the influence of the heating holder with different supporting membrane materials—C or Si nitride (SiN_x_)—on Si NW morphology was studied.

## 2. Experiments

To prepare Si NW samples for in situ TEM characterization, Si NWs grown on a glass substrate were harvested by dipping them into an ethanol solution with the help of an ultrasonic bath system. Then, an ethanol droplet containing Si NWs was transferred with a pipette from the solution to the specific TEM Protochips Aduro heating chip coated with a C membrane or a SiN_x_ membrane. It should be noted here that there are only three types of membranes for heating chips from Protochips, including SiC, C, and SiNx. The maximum temperature allowed by the system is 1200 °C, with good heat uniformity and 5% temperature accuracy. Next, the sample holder with the chip was loaded in a JEOL 2010F operated at 200 kV and 10 nA. It should be noted here that there are two reasons for choosing 10 nA as the working current: Firstly, because this is the current used in standard in situ TEM experiments for the sake of visibility. Secondly, because it delivers clear facts when one wants to find evidence of beam damage. The TEM image acquisition time was 0.5 s, except when mentioned otherwise. It is reported in the literature that the melting behavior of nanostructures is strongly dependent on their size, i.e., the melting temperature of Si NWs decreases with the reduction in their diameter [38]. In order to study the diameter dependence of electron irradiation on the Si NWs, Sn was used as a catalyst instead of Au, since Sn-catalyzed Si NWs exhibit strong tapering (i.e., the diameter of the Si NWs becomes thinner as it gets closer to the NW top end), unlike the cylindrical morphology of Si NWs catalyzed by Au. Growth conditions resulting in tapered NWs can be found in our previous work [39].

## 3. Results and Discussion

**Figure 1:**
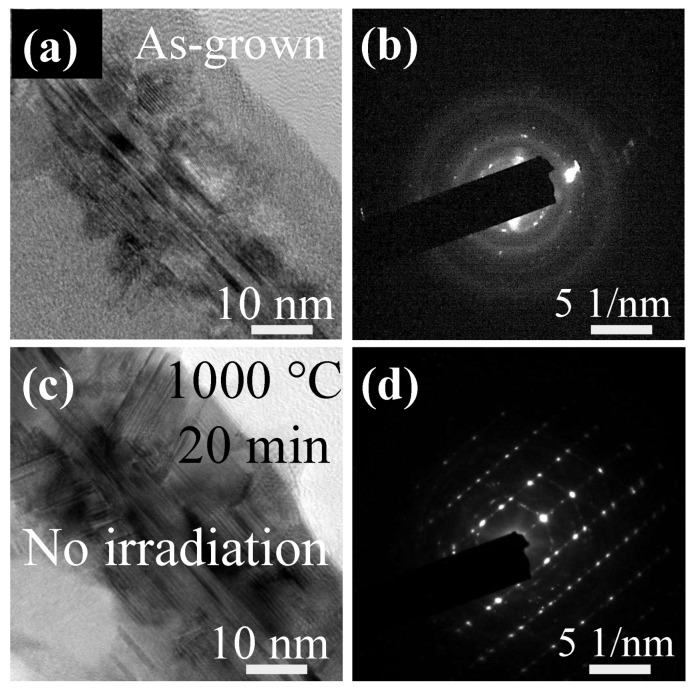
(**a**) Middle zone of an as-grown Si NW, and (**b**) its corresponding diffraction pattern. (**c**) The same Si NW as in panel (**a**), but annealed at 1000 °C for 1200 s without beam irradiation, and (**d**) the corresponding diffraction pattern.

The evolution of Si NWs on the C membrane during in situ TEM heating was studied. First of all, pure thermal heating of Si NWs was investigated. Note that heating was performed in the TEM chamber, but without exposure to the e-beam. The TEM image in Figure 1a shows the middle zone of an as-grown Si NW that has not been exposed to the e-beam, with its corresponding diffraction pattern (DP) presented in Figure 1b. The TEM image acquisition time in Figure 1b was 5 s. Its structure consists of a crystalline core and an amorphous shell. This core–shell structure is due to the growth of the NW core with the mediation of a metal droplet, along with the deposition of an a-Si: H shell on the NW sidewall during NW growth [39]. When a Si NW with such a core–shell structure undergoes heating at 1000 °C for 1200 s, a phase transformation of the shell from amorphous to crystalline can be observed, as evidenced by TEM in Figure 1c, with the corresponding diffraction pattern in Figure 1d. The TEM image acquisition time in Figure 1d was 3 s.

**Figure 2:**
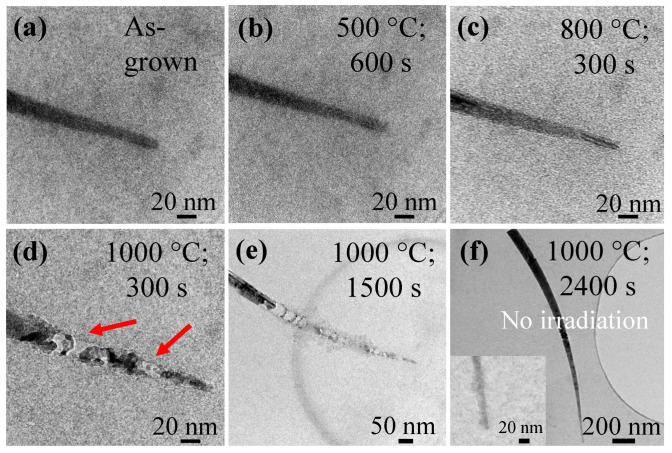
In situ TEM observation of Sn-catalyzed PECVD-grown Si NWs: (**a**) Top end of as-grown Si NW transferred onto an a-C membrane. (**b**–**e**) Heating of the Si NW at different temperatures and for various durations. (**f**) Si NW after heating at 1000 °C for 2400 s without exposure to electron irradiation. Inset showing the enlarged view of the NW tip. The red arrows in (**d**) show the degradation regions.

Let us now evaluate the effects of e-beam irradiation on the NW structure. Note that the electron flux is around 7000 electrons/s/Å^2^ for Figure 2b–d, while it is 670 electrons/s/Å^2^ for Figure 2e. It can be seen from Figure 2a (as-grown) and Figure 2b (dose at ≈ 4.24 × 10^6^ electrons/Å^2^) that no evolution of the Si NW can be observed when the heating is performed at a temperature of 500 °C for 600 s. Now, raising the heating temperature to 1000 °C and keeping it for 300 s (dose at 2.12 × 10^6^ electrons/Å^2^) and 1500 s (dose at 1.01 × 10^7^ electrons/Å^2^), as shown in Figure 2d,e, respectively, amplifies the degradation. Comparing Figure 2c,d, a conclusion can be made that, unsurprisingly, higher temperatures accelerate degradation after the same irradiation dose and time. In Figure 2e, the irradiated part of the Si NW annealed at 1000 °C is obviously degraded as compared to the one without exposure to the e-beam, as shown in Figure 2f. In Figure 2f, there is no formation of SiC without the beam when the temperature is maintained at 1000 °C, even for 2400 s. Note that there is a thin native oxide layer on the Si NWs, since no HF dipping is applied to the Si NWs before loading them into the TEM chamber. The recrystallization of the entire NW shell to single-crystalline in Figure 1c indicates that the covered native Si oxide is very thin. However, the thickness of this native oxide is not uniform. The degradation of Si NWs would start earlier at the region of the thinner native oxide layer. As a result, the inhomogeneous degradation of NWs occurred.

**Figure 3:**
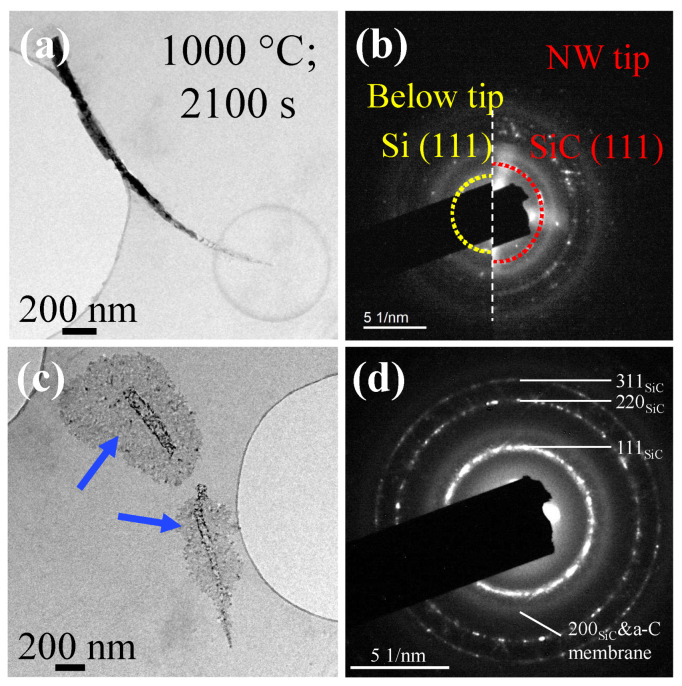
(**a**) Low-magnification image of a Si NW heated at 1000 °C for 2100 s. (**b**) DPs at the NW tip and below the tip. (**c**) Low-magnification image of a Si NW after heating at 1100 °C for 600 s, and then 1200 °C for 300 s, under no irradiation. (**d**) DP of the area. The blue arrows in (**c**) show the formation of β-SiC in the non-irradiated areas.

To investigate whether there is chemical evolution of Si NW upon irradiation annealing, the DPs of Si NWs were studied. The analyzed Si NW (heated at 1000 °C for 2100 s, with electron flux at 100 electrons·s^−1^·Å^−2^) is presented in Figure 3a, with the DPs of the NW tip and below the NW tip (no degradation zone) shown at the right part and the left part in Figure 3b, respectively. The DP of the NW tip indicates newly formed rings at the NW tip, which can be indexed in terms of β-Si carbide (SiC). Thus, electron irradiation triggers the formation of SiC at 1000 °C in the vicinity of the NW, probably by pushing Si surface atoms into the nearby amorphous C membrane. Electron sputtering of Si surface atoms is indeed very efficient at 200 keV [40]. We then heated the C membrane up to 1200 °C—the maximum temperature allowed by the system. This caused the formation of β-SiC to spread to non-irradiated areas (Figure 3c), as shown by the blue arrows. All rings of DP (Figure 3d) can be interpreted in terms of β-SiC or amorphous C membrane; all of the Si NWs were converted into SiC nanoparticles. These experiments thus reveal a chemical interaction of Si NWs with the C membrane due to a combination of electron beam and thermal effects.

**Figure 4:**
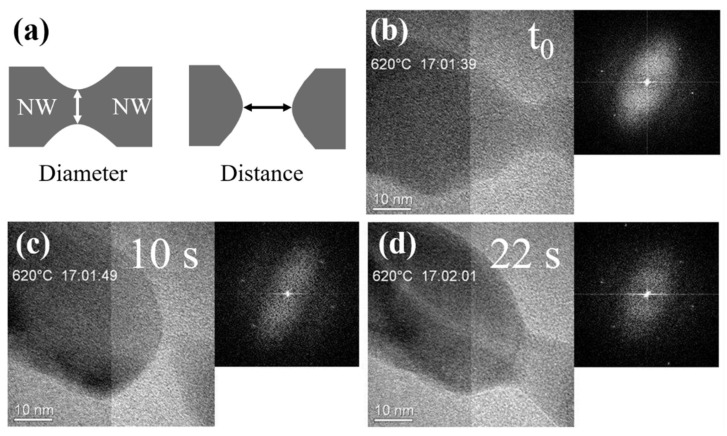
(**a**) Schematic illustration of NWs showing the regions where diameter and distance are measured. Creep of a single Si NW under the beam during continuous heating at 620 °C with the heating time of (**b**) *t*_0_, (**c**) 10 s, and (**d**) 22 s. (**a**–**c**) NW cutting; (**d**) NW welding. Insets represent the corresponding Fourier transforms.

To avoid the interaction between the supporting membrane and the Si NWs, we then used heating chips equipped with a SiN_x_ membrane. In this case, we still obtained very significant changes in the shape of the NWs under irradiation at 620 °C, as presented in Figure 4. The sequences of TEM images in Figure 4 were extracted from Video S1. At *t*_0_ (Figure 4b), considered as the starting point of investigation, we can see that a strong creep of Si NW occurred. After 10 s, a crack in the Si NW could be observed (Figure 4c). Interestingly enough, this was followed by the welding of Si NW at the crack region, as presented in Figure 4d. The two NW parts could be separated and welded consecutively into one single Si NW. The corresponding Fourier transforms in the insets indicate that the crystalline structure of Si NW is maintained during the evolution of NW morphology.

**Figure 5:**
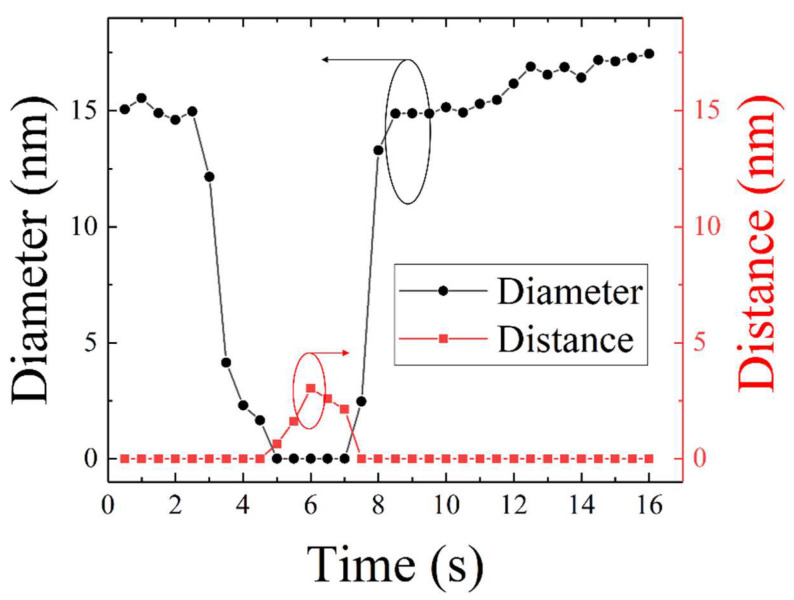
Evolution of NW diameter at the crack area, and distance between two separated NW parts as a function of heating time. See the representative illustration of diameter and distance in Figure 4.

To quantitatively analyze the changes in NW morphology as a function of heating time, their diameter at the narrow point (black circles) and the distance between the two parts (red squares) were measured continuously with a time step of 0.5 s, as presented in Figure 5. Note that the diameters and distances were taken as illustrated in Figure 4a. It can be seen from Figure 5 that the diameter decreased rapidly after 2.5 s of heating, and the crack occurred when the heating time reached 4.5 s. Then, the distance between the two separated NW parts increased gradually until it reached the maximum value of 3 nm at 6 s. After that time, the distance decreased with the increase in heating time, suggesting that the welding of two separated parts began until they were fused together at 7.5 s. The diameter after fusing increased dramatically in the first 1 s, but then increased slowly. It can be seen from Figure 5 that the cracking of the NW under e-beam heating is reversible. This interesting phenomenon suggests that the beam can be used in the healing of nano-objects.

The influence of the e-beam on the analyzed Si NW samples can be discussed by considering three effects of the TEM electron beam: First of all, heating by the e-beam results in the inelastic scattering of electrons. Moreover, the diameter of the NW tip is below 20 nm, which is smaller than the inelastic mean free path of electrons (the value of which is calculated below). The maximum temperature increase ΔT of the NW, without considering the radiative emission from NW and substrate, is given as follows [41]:

(1) $$\Delta T=\frac{I\langle E\rangle }{4\pi \kappa e\lambda }\left[0.58+2\ln\left(\frac{2R_{0}}{d}\right)\right]$$

where I is the beam current, 〈E〉 is the average energy lost by electrons after inelastic collision, κ is the thermal conductivity of the material, e is the elementary charge, and λ is the inelastic mean free path of electrons, given as follows [42]:

(2) $$\lambda^{-1}=\frac{1}{{\pi a}_{0}v^{2}}\left[A\;\mathrm{In}\left(\frac{2v^{2}}{I_{x}}\right)-\frac{7C}{v^{2}}\right]$$

where $a_{0}$ is the Bohr radius, and the quantities A, $I_{x}$, and C are material parameters. The parameter $I_{x}$ has units of energy, but its physical nature is still unknown; v is the electron velocity, $v^{2}=\frac{c^{2}\tau \left(\tau +2\right)}{{\left(\tau +1\right)}^{2}},\;\tau =\frac{V}{c^{2}}$, with values of $\hbar =m_{e}=e=1,$ c is the velocity of light ($c^{2}\approx 510999\;\mathrm{eV}$), V is the incident electron energy in eV, d is the diameter of the electron beam, and $R_{0}$ is the reference radius with respect to the temperature difference. Note here that Equation (1) models a two-dimensional (2D) homogeneous system, but the electron beam indeed is much wider; therefore, Equation (1) is appropriate for our sample. For electrons with energy greater than 50 keV, the range follows a power law [43]:

(3) $$R_{0}\left(V\right)={\mathrm{bV}}^{n}\left[1-\frac{1}{{\left(1+\frac{V}{{\mathrm{Nm}}_{e}c^{2}}\right)}^{2}}\right]$$

where we take into account relativistic corrections. The numbers b, n, and N are fitting parameters. Let us now consider the maximum heating induced by the beam in Si. For Si, we have b = 0.542 µm·eV^−n^, n = 0.676, and N = 5 [43], and the beam current I is close to 10 nA.

The energy lost by the electron can be transferred to a phonon, plasmon, or another electron in the electronic shell of an atom. The average energy lost by electrons due to inelastic scattering 〈E〉 depends on the material that is crossed. Let us assume that all of the incident electrons transfer a part of their kinetic energy to other electrons, promoting them to a higher energy level. In Si, the energy transfers recorded by electron energy-loss spectroscopy (EELS) drastically drop above 100 eV, which corresponds to the L transition [44]. The maximum heating can be estimated by taking 〈E〉 = 100 eV. The thermal conductivity κ of Si is 150 W·m^−1^·K^−1^ [45]. For an incident electron energy of 200 keV, the parameters of A (eV), I_x (eV), and C (eV^2^) are 26.42 eV, 25.55 eV, and 1495.47 eV^2^, respectively, in Si, and the inelastic mean free path λ is 157.76 nm [41]. At our working magnification, sufficient to gather information on the nanowire crystalline structure, the probe diameter d is 70 nm. Putting these numbers into Equation (1), with R_0,Si_ = 290 µm, gives a maximum heating of 0.06 K. This value is most likely overestimated, since we only considered one channel for incident electrons to transfer their kinetic energy. Other channels, such as plasmon excitations, result in a much lower energy loss—in the 50 eV range—and have much higher cross-sections. The final conclusion is that the heating effect of the electron beam is not sufficient to change the growth dynamics. Therefore, the morphological and structural evolution of our samples caused by beam irradiation via heating effects can be excluded. Heat caused by the electron beam can be conducted along NWs as well on substrates. Indeed, we took into account the contribution of the substrate to the heat conduction. We calculated the R_0_ of the C-membrane substrate to be 252 μm, which is smaller than the R_0_ of Si (290 μm). The smaller value of R_0_ means a lower temperature increase. The purpose of this discussion being to evaluate the maximum temperature increase that can be achieved in the NWs, we adopted the larger R_0_. Of course, introducing radiative losses would further decrease the heating; thus, neglecting it does not invalidate our conclusion.

Secondly, high-energy electrons can induce a knock-on effect, resulting in the displacement of atoms. The minimum incidence-electron energy can be written as follows [46]:

(4) $$V_{\max}=\left(m_{0}c^{2}\right)\cdot (\sqrt{1+\frac{{\mathrm{AE}}_{d}}{561\;\mathrm{eV}}}-1)$$

where $m_{0}c^{2}=511000\;\mathrm{eV}$, and the displacement energy $E_{d}$ of a Si atom is 4.63 eV. Therefore, according to Equation (4), the threshold energy that allows a knock-out effect is 56.17 keV. This value is smaller than the incident electron energy of 200 keV used in this work. Therefore, e-beam sputtering of Si atoms should be included.

The third effect caused by the e-beam is radiolysis, which is achieved by breaking atomic bonds. As far as the heating of Si NWs is considered, pure thermal heating can crystallize the a-Si: H shell and result in a fully crystallized NW. However, degradation occurs when heating is performed under e-beam irradiation. It can be seen from Figure 3a that the degradation of the NW does not start at the tip. With the irradiation time increasing, degradation spreads on both sides of the original vanishing parts. 

## 4. Conclusions

In summary, by comparing the heating of an individual Si NW placed on a C membrane under the same temperature, with or without beam irradiation, we were able to analyze the beam’s effects on Si NW annealing. The degradation of NWs starts near the tip, where they are thick enough for the electron interactions to build up. When the supporting membrane is made of SiN_x_, no interaction between the membrane and the NWs occurs. At 620 °C, the creep of Si NWs is observed, with the formation of a crack after 4.5 s. Surprisingly, the sectioning of Si NWs is reversible via the welding of two separated parts at 7.5 s. In situ TEM experiments are now developing rapidly; by describing effects essentially due to irradiation, rather than to the imposed high temperature, the present experiments give important information on the beam’s effects, which will be useful for many researchers.

## Data Availability

Not applicable.

## Supplementary Materials

The following supporting information can be downloaded at: https://www.mdpi.com/article/10.3390/ma15155244/s1, Video S1: The sequences of TEM images of the Si NWs heated at 620 °C for 17 s.
